# In vitro myotoxic effects of bupivacaine on rhabdomyosarcoma cells, immortalized and primary muscle cells

**DOI:** 10.1186/s12935-015-0229-6

**Published:** 2015-07-29

**Authors:** Thomas Metterlein, Petra Hoffmann, Ruth Späth, Michael Gruber, Bernhard M Graf, Wolfgang Zink

**Affiliations:** Department of Anesthesiology, University Hospital Regensburg, 93051 Regensburg, Germany; Department of Anesthesiology and Intensive Care Medicine, Klinikum Ludwigshafen, Ludwigshafen, Germany

**Keywords:** Myotoxicity, Rhabdomyosarcoma, Bupivacaine, Cell culture

## Abstract

**Background:**

Rhabdomyosarcoma is a rare malignant skeletal muscle tumor. It mainly occurs in children and young adults and has an unsatisfactory prognosis. Prior studies showed a direct myotoxic effect of bupivacaine on differentiated muscle cells in vitro and in vivo. Exact mechanisms of this myotoxicity are still not fully understood, but a myotoxic effect on malignant muscle tumor cells has not been examined so far. Thus, the aim of this study was to examine if bupivacaine has cytotoxic effects on rhabdomyosarcoma cells, immortalized muscle cells and differentiated muscle cells.

**Methods:**

Cell lines of rhabdomyosarcoma cells, immortalized muscle cells and differentiated muscle cells were established. After microscopic identification, cells were exposed to various concentrations of bupivacaine (500, 1,000, 1,750, 2,500 and 5,000 ppm) for 1 and 2 h, respectively. 24 and 28 h after incubation the cultures were stained with propidium iodid and analyzed by flow cytometry. The fraction of dead cells was calculated for each experiment and the concentration with 50% cell survival (IC50) was computed. Cell groups as well as incubation and recovery time were compared (ANOVA/Bonferroni p < 0.01).

**Results:**

The total number of cultured cells was similar for the different local anesthetics and examined concentrations. Increasing concentrations of bupivacaine led to a decrease in survival of muscle cells. IC50 was highest for immortalized cells, followed by rhabdomyosarcoma cells and differentiated cells. Exposure time, but not recovery time, had an influence on survival.

**Conclusion:**

Bupivacaine has clear but different cytotoxic effects on various muscle cell types in vitro. Differentiated primary cells seem to be more vulnerable than tumor cells possibly because of more differentiated intracellular structures.

## Background

Rhabdomyosarcoma is a rare (4–7/1,000,000 children) and serious childhood cancer entity that arises from primitive muscle cells, the “rhabdomyoblasts”. The tumor cells fail to differentiate into adult striated muscle cells. These tumors account for about 5–8% of all childhood cancers, with a peak incidence in the age group of 1–5 years. Overall, 50% of the children diagnosed with rhabdomyosarcoma survive 5 years. If possible radical resection and adjuvant radio-chemotherapy is performed. Patients with metastatic disease have a poor prognosis because specific chemotherapeutic approaches are missing [1].

Myotoxic properties have been described for various local anesthetics, with bupivacaine apparently being most myotoxic [2]. Exact mechanisms of the cytotoxic effect on skeletal muscle cells are still not entirely understood. However, an involvement of intracellular calcium homeostasis has been shown to play an important role [3]. Local anesthetics (LA) increase intracellular calcium levels by inducing sarcoplasmic release and simultaneously inhibiting calcium reuptake into the sarcoplasmic reticulum [3]. Furthermore inhibition of mitochondrial function with consecutively impaired cellular energy balance has also been described. Additionally to immediate necrosis of myocytes, certain LAs can induce apoptosis [3, 4]. Prior studies could show that apoptosis occurs within a few hours after treatment with local anesthetic. After 24 h mainly late apoptosis and necrosis can be found. At this stage cells can be identified by staining with propidium iodid (PI) [5].

The clinical effects of local anesthetic induced myotoxicity often remain unrecognized. The affected muscle recovers within weeks after local anesthetic induced damage. Histological studies show that cell debris is rapidly removed. Damaged muscle cells are replaced by myoblasts that divide and form myotubes. Finally, the growing cells merge and differentiate into adult skeletal muscle fibers. To allow this recovery, undifferentiated myoblasts are suggested to be resistant to local anesthetics. Rhabdomyosarcomas arise from undifferentiated myoblasts and might therefore also be resistant to local anesthetics.

Rapidly dividing cancer cells show numerous differences from differentiated cells of the same tissue type. In order to divide, the cell cycle is steadily repeated, with a concomitant doubling of macromolecular content. Rhabdomyosarcomas develop from myoblasts and fail to differentiate due to its rapid growth at the cost of differentiation [6].

Commercially available immortalized cell lines are often used for experiments because they are easier to grow and often more robust to external stimuli. Morphologically these cells are not different from the original tissue but functional modifications are possible. A suppression of apoptotic pathways is often seen.

Myotoxic properties of bupivacaine on differentiated muscle cells as well as immortalized cells can be studied in cell cultures [4]. Rhabdomyosarcoma cells easily grow in vitro allowing the examination of potentially toxic substances [7].

Aim of this specific study was to investigate potential differences in cytotoxic effects of bupivacaine on rhabdomyosarcoma cells, immortalized muscle cells and differentiated muscle cells in vitro.

## Methods

### Cell culture

Rhabdomyosarcoma cells (CLS-Cell Lines Service, Eppelheim, Germany), immortalized C2C12 (CLS-Cell Lines Service, Eppelheim, Germany) muscle cells and primary muscle cells were used for the study.

### Cell line establishment

Cells were grown and differentiated as described earlier. [7] The C2C12 and the rhabdomyosarcoma cell line was grown in Dulbecco’s modified Eagle’s medium (DMEM) (PAA Laboratories GmbH, Pasching, Austria) supplemented with 10% fetal bovine serum (Sigma-Aldrich Chemie GmbH, Taufkirchen, Germany) and l-glutamine 2 mM (Life Technologies GmbH, Darmstadt, Germany).

The primary muscle cell culture was established with consent of the local committee for Laboratory Animal Care. Muscle cells of BALB/c AnNcrl mice were extracted, prepared and washed in phosphate buffered saline (PBS) (Sigma-Aldrich Chemie GmbH). Intact muscles were incubated for 1.5 h with 8 mL 0.2% Collagenase Typ 1 (GIBCO^®^ Cell Culture, Invitrogen GmbH, Darmstadt, Germany) in DMEM, 1% penicillinstreptomycin (Sigma-Aldrich Chemie GmbH) and 1% l-glutamine (Sigma-Aldrich Chemie GmbH) in Petri dishes (Sigma-Aldrich Chemie GmbH). Skeletal muscles were carefully dissolved, and myofibers were separated. Intact single fibers were brought onto Matrigel and DMEM-coated 6-well plates. After 3 min, 0.5 mL of plating medium (Table 1) was added. After 3 days, plating medium was replaced by proliferation medium (Table 1).

**Table 1:** Different types of medium for primary skeletal muscle cell culture

Plating medium	Dulbecco’s modified Eagle’s medium (DMEM) 10% horse serum (HS) 0.5% chick embryo extract (CEE) 1% penicillin–streptomycin (P/S) 1% l-glutamine
Proliferation medium	DMEM 10% HS 10% fetal bovine serum (FBS) 1% CEE 1% P/S 1% l-glutamine
Differentiation medium	DMEM 2% FBS 0.5% CEE 1% P/S 1% l-glutamine

**Fig. 1:**
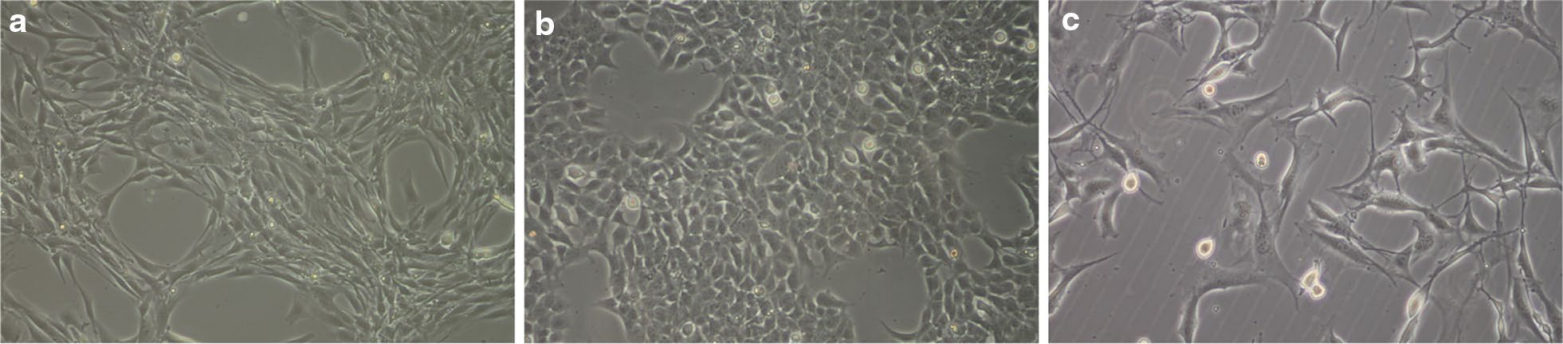
Microscopic images of **a** C2C12 cells; **b** primary muscle cells and **c** rhabdomyosarcoma cells.

Having established a confluent monolayer in the cell lines after 3 days, medium was changed into differentiation medium (Table 1) to force myoblasts to differentiate into myotubes (Fig. 1).

Cells were harvested with Trypsin–EDTA (Sigma-Aldrich Chemie GmbH) diluted 1:4 with PBS without calcium and magnesium chloride (Sigma-Aldrich Chemie GmbH).

### Cell treatment

For the actual study cells were removed from the primary flask, counted and transferred to 6-well-plates (neoLab Migge Laborbedarf-Vertriebs GmbH, Heidelberg, Germany) with approximately 150,000 cells per well. 72 h after dissemination, cells were treated with bupivacaine hydrochloride (Sigma-Aldrich Chemie GmbH, Taufkirchen, Germany) in concentrations of 0, 500, 1,000, 1,750, 2,500 and 5,000 ppm. Every treatment was done in duplicate (two wells) at each of the three series. After incubation for 1 and 2 h bupivacaine was removed and cells were washed with PBS and cultured with growth medium, described above, for another 24 and 48 h recovery time. Because evaluation was accomplished immediately after staining no fixation occurred. The cells from every harvest well were measured separately.

### Cytotoxicty evaluation by flow cytometry

Necrotic cells without intact cell membrane were marked with PI (15 µM) (Invitrogen GmbH, Darmstadt, Germany) and counted by flow cytometry (FACS Calibur, Becton–Dickinson, Heidelberg, Germany) The fraction of dead cells in relation to total number of cells was calculated by counting a total of 5,000 events per well.

### Statistical analysis

For each cell type, incubation and recovery time the theoretical concentration with 50% cell survival (IC50) was calculated. For this pharmacodynamic modeling and analysis Phoenix™WinNonlin^®^ 6.2 (Pharsight, Certara, St. Louis, MO, USA) was used. IC50 were compared between cell types, incubation and recovery time using an ANOVA with Bonferroni correction with p < 0.01 considered significant. Statistical analysis was accomplished with Microsoft Excel and IBM SPSS Statistics Version 19 (IBM Deutschland GmbH, Ehningen, Germany).

## Results

### Cell line identification

The growing cells could be identified microscopically according to morphological characteristics as rhabdomyosarcoma cells or differentiated muscle cells (Fig. 1).

### Cell death measurement

**Fig. 2:**
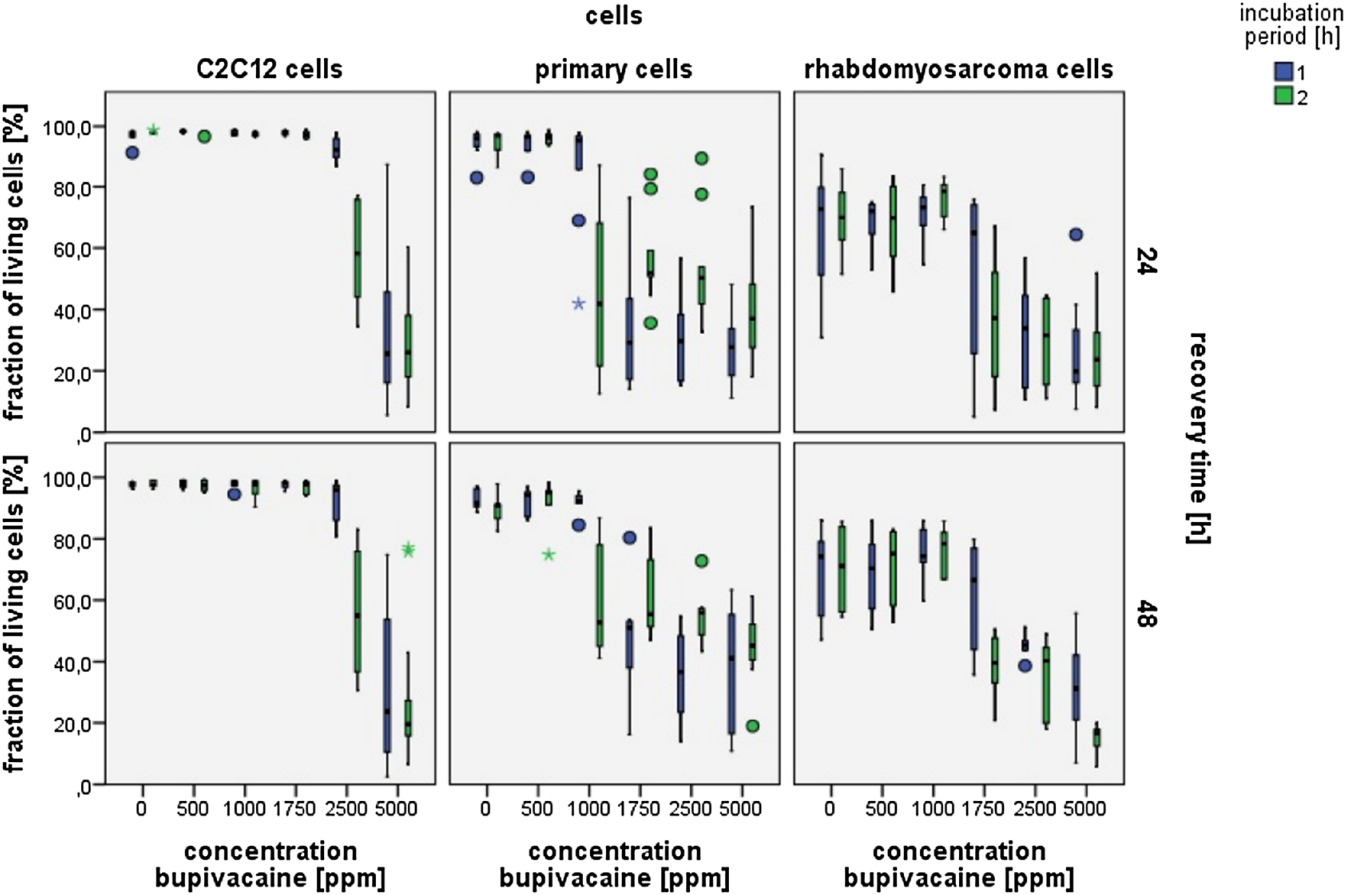
Fraction of living cells 24 and 48 h after incubation with increasing bupivacaine concentrations. Two different incubation periods are shown. Values are given as medians. 75% percentiles, minimum and maximum.

Cell death was dose-dependently induced in primary muscle cells, immortalized muscle cells and rhabdomyosarcoma cells. Results are given in Fig. 2.

### Calculated IC50 values

Pharmacodynamic modeling showed different IC50 concentrations for the investigated cell lines, incubation and recovery periods (Table 2). The IC50 was highest for immortalized C2C12 cells followed by rhabdomyosarcoma cells and differentiated primary muscle cells at the same incubation and recovery time.

**Table 2:** Calculated bupivacaine concentration (ppm) with 50% cell survival (IC50)

Incubation/recovery time (h)	Differentiated primary muscle cells [IC50 ± SD] (ppm)	Rhabdomyosarcoma cells [IC50 ± SD] (ppm)	Immortalized C2C12 cells [IC50 ± SD] (ppm)
1/24	1,232 ± 421	2,400 ± 703	3,814 ± 1,104
1/48	1,472 ± 438	1,908 ± 658	3,436 ± 1,266
2/24	834 ± 300	1,958 ± 620	2,413 ± 840
2/48	1,132 ± 442	1,871 ± 598	2,526 ± 826

For all cell types 2 hours incubation lead to more dead cells than incubation for one hour.

For a given cell type and incubation period recovery time did not influence the fraction of dead cells.

Combined results ignoring incubation and recovery time showed a significantly different behavior of the examined cell lines. With more cell survival for immortalized cells, followed by rhabdomyosarcoma cells and primary muscle cells (Table 3).

**Table 3:** Combined calculated IC50 bupivacaine concentrations (ppm) differentiating only cell type

Incubation/recovery time combined (h)	Differentiated primary muscle cells [IC50 ± SD] (n = 24)	Rhabdomyosarcoma cells [IC50 ± SD] (n = 31)	Immortalized C2C12 cells [IC50 ± SD] (n = 24)
	1,161 ± 403* ppm	2,034 ± 584* ppm	3,297 ± 1,247* ppm

ANOVA with Bonferroni * p < 0.01.

## Discussion

According to both in vitro and in vivo studies, local anesthetics have myotoxic properties. Various mechanisms of this myotoxicity are discussed. Local anesthetics influence the cellular calcium homeostasis, energy balance and liberate intracellular free radicals capable of inducing apoptotic cascades. Prior investigations revealed that bupivacaine seems to be the most myotoxic local anesthetic of all clinically used substances [8].

Histological studies show a mixed picture of cell damage after exposure to bupivacaine. Morphologically sarcolemma membranes remain intact until total lysis and fragmentation of the fibers occurs. Vasculature, neural structures and connective tissue elements are visibly not affected in the examined sections [9]. Clinically, affected muscles rapidly start to recover after exposure to myotoxic local anesthetics. Histological studies show that myoblasts start to divide and transform after elimination of cell debris. Growing myotubes elongate and eventually merge into adult muscle cells [4]. Myoblasts as undifferentiated precursor cells therefore serve as an important reservoir for damage repair. To allow this form of recovery undifferentiated myoblasts have to be less vulnerable to anesthetic-induced damage.

The results of our in vitro study show that all examined cells are vulnerable to increasing concentrations of bupivacaine. Primary differentiated muscle cells are more vulnerable than tumor cells and immortalized muscle cells.

An important difference between mature myocytes and myoblasts is the differentiation with highly specific calcium storage and release mechanisms. An important mechanism of local anesthetic induced myotoxicity seems to be a pathological influence on the cellular calcium homeostasis. An increased sarcoplasmic calcium release via the specific ryanodine receptor can be demonstrated in vivo [6]. Local anesthetics also decrease the sarcoplasmic calcium reuptake by blocking the sarcoplasmic calcium ATPase (SERCA) [3]. Due to the more developed calcium handling mechanisms differentiated myocytes are more vulnerable to local anesthetics.

Human rhabdomyosarcoma cells develop from myoblasts and fail to differentiate into adult muscle cells. The rapid growth is at the cost of differentiation [6]. Tumor cells are lacking a contractile apparatus with the accompanying calcium stores [3]. Calcium mediated myotoxic effects are therefore less likely in sarcoma cells. However this study shows that bupivacaine induces cell death in human rhabdomyosarcoma cells in vitro.

A possible explanation might be an affected cellular energy balance. Local anesthetics can influence cellular energy production by blocking mitochondrial function [10]. Sarcoma cells, similar to all fast growing tumor cells, have an increased energy demand. Compared to resting myoblasts sarcoma cells are rapidly dividing. This involves increased energy consumption [11]. An acute disruption of the cells mitochondrial energy supply could be compensated in a resting cell but not in cells with already increased energy demand. Any problem in the energy supply can lead to a complete breakdown of the cellular homeostasis.

Another possible pathway for bupivacaine induced toxic effects on sarcoma cells is the induction of apoptosis via activation of various caspases [12–14]. Immortalized muscle cells are modified to evade apoptosis. This could explain why these cells seem to be more resistant to bupivacaine. Immortalized muscle cells morphologically look like differentiated muscle cells but functionally behave differently. A less developed calcium handling apparatus and suppressed apoptotic pathways make these cells less vulnerable to external stimuli. A behavior intended in commercially available cell lines.

As seen in prior laboratory and clinical studies incubation time had an effect on cell survival. The longer the cells were exposed to with myotoxic substance, the more severe was the damage [7].

According to this study, recovery time had no effect on the fraction of cell survival. Surviving cells usually resume replication within hours and the amount of vital cells should increase with recovery time [11]. However, in the additional 24 h of recovery, cells did not multiply at rates seen without local anesthetic intoxication. The induced cytotoxic effects are apparently longer lasting than the actual incubation. Sustained inhibitory effects on cell replication or delayed cytotoxic mechanisms might be a possible explanation and need to be further investigated.

## Conclusion

In vitro differentiated primary muscle cells are more vulnerable to bupivacaine than rhabdomyosarcoma cells and immortalized muscle cells. Exact mechanisms of this cytotoxicity are unknown and subject to further studies. Whether this described behavior is reproducible in vivo is subject to further research.

## Abbreviations

PI: propidium iodid
LA: local anesthetic
PBS: phosphate buffered saline
SERCA: sarcoplasmic calcium ATPase
IC50: concentration with 50% cell survival
ANOVA: analysis of variance
SD: standard deviation
ppm: parts per million
